# Robustness of Self-Organised Systems to Changes in Behaviour: An Example from Real and Simulated Self-Organised Snail Aggregations

**DOI:** 10.1371/journal.pone.0022743

**Published:** 2011-07-28

**Authors:** Richard Stafford, Gray A. Williams, Mark S. Davies

**Affiliations:** 1 Luton Institute of Research in Applied Natural Sciences, University of Bedfordshire, Luton, United Kingdom; 2 The Swire Institute of Marine Science and School of Biological Sciences, The University of Hong Kong, Hong Kong; 3 Faculty of Applied Sciences, University of Sunderland, Sunderland, United Kingdom; Cajal Institute, Consejo Superior de Investigaciones Científicas, Spain

## Abstract

Group or population level self-organised systems comprise many individuals displaying group-level emergent properties. Current theory indicates that individual-level behaviours have an effect on the final group-level behaviour; that is, self-organised systems are sensitive to small changes in individual behaviour. Here we examine a self-organised behaviour in relation to environmentally-driven individual-level changes in behaviour, using both natural systems and computer simulations. We demonstrate that aggregations of intertidal snails slightly decrease in size when, owing to hotter and more desiccating conditions, individuals forage for shorter periods – a seemingly non-adaptive behaviour for the snails since aggregation reduces desiccation stress. This decrease, however, only occurs in simple experimental systems (and simulations of these systems). When studied in their natural and more complex environment, and simulations of such an environment, using the same reduced foraging time, no difference in aggregation behaviour was found between hot and cool days. These results give an indication of how robust self-organised systems are to changes in individual-level behaviour. The complexity of the natural environment and the interactions of individuals with this environment, therefore, can result in self-organised systems being more resilient to individual-level changes than previously assumed.

## Introduction

Self-organised systems are frequently considered sensitive to changes in the individual-level behaviours of organisms that comprise the systems [1], [2]. For example, studies on simulations of army ants have demonstrated that certain parameters, such as individual variability in direction of movement, greatly affect the colony's foraging efficiency, and that in nature, these ‘parameters’ or behaviours are optimally set to maximise foraging [1]–[5].

The rocky shore habitat experiences very clear environmental changes with the rise and fall of the tide, and species in this habitat are easy to study and manipulate in field or laboratory experiments. As a result, rocky shore species are ideal candidates to examine how environmental variables affect individual-level behaviours [6]–[8]. For example, increasing air temperature when mobile invertebrates are emersed results in decreased foraging times (as a result of greater desiccation stress) [e.g. 9], [10], resulting in animals becoming inactive, often in groups, when the tide is out. Often, animals try to find shelter from the desiccating conditions associated with emersion, and can be found in cracks and crevices in the rock surface, or in aggregations [10]. Self-organisation of aggregation behaviour has been documented in species of high-shore littorinid snails [11]–[13], and simulation models of these snails have been validated on data collected from natural shores [12]. This system, therefore, provides an excellent opportunity to test predictions on naturally-occurring changes in individual-level behaviour, and the resultant, group-level, self-organised distributions that form under different environmental conditions. In this study we test the effects of increased desiccation stress on the ability of snails to form aggregations, using natural systems (rocky shores on hot and cool days), simplified experimental treatments (artificial rock slabs with roofs to decrease desiccation stress), and computer simulations of the natural and experimental systems.

## Methods

### Study system

In Hong Kong, during the incoming tide, high-shore littorinid snails (*Echinolittorina malaccana* and *E. radiata*) move up the shore to feed in the awash zone (wet by the waves, but not submerged) [10], [12]. As the tide retreats, the snails move back down the shore and form aggregations of 2 to >50 individuals as the rock surface dries [10], [12]. Aggregations reduce desiccation [10] and this behaviour is important because snails can be emersed for >24 h between tides that are high enough to wet them [10], [12].

Snails show three simple behaviours that, when simulated, account for their distribution patterns. Firstly, if snails encounter another individual (by chance), then they can choose to stop and aggregate. Secondly, if snails encounter a crevice (a depression in the rock that they can enter), they can choose to remain in the crevice. Thirdly, snails can follow trails of mucus, laid previously by other individuals (of either species) on the substratum and which persist over tidal cycles. Using only these three ‘rules’, the simulated snails show the same spatial distribution patterns as real snails [12]. Aggregations, therefore, result from a process of self-organisation. Essentially aggregations arise from chance encounters with other individuals, but some of these chance encounters are facilitated by the following of mucus trails. Thus likely sites of large aggregations are where trails cross each other, or where trails cross crevices or other topographic features causing ‘trail leaders’ to slow or stop [12].

### Natural shore study

The percentage of snails aggregating during an ebbing tide after high water was investigated at two shores on Hong Kong Island, Cape d'Aguilar (22.3037°N, 114.2558°E) and Wah Fu (22.2499°N, 114.1304°E), in August and September 2001. The percentage of snails in aggregations (aggregations defined after [10], [14], [15] as ≥3 individuals in direct physical contact with each other) was calculated in ten, randomly located, quadrats (0.25 m^2^) along a pre-determined 50-m stretch of shore. Sampling was repeated on three replicate days under two different sets of environmental conditions: Hot Days (H, air temperature 300 mm above the rock surface was >35°C) and Cool Days (C, temperature was <28°C). Other factors, such as wave action, were as similar as possible between the different days, although wave action was slightly lower at Cape d'Aguilar (<100 mm high waves) than at Wah Fu (300–500 mm, predominantly due to passing boat traffic). The order in which the samples were taken at each shore was randomly determined, with at least 48 h separating replicate sample days. Between sample days, snails were seen actively foraging during high tide, confirming that individuals moved between sample days. To confirm the assumed increase in desiccation stress on Hot as compared to Cool Days, cotton wool balls soaked in water (n = 5) were weighed before and after 1 h of emersion on the rock surface at Cape d'Aguilar on each of the replicate days, and the percentage water loss calculated.

### Semi-artificial environment study

Although we attempted to minimise possible confounding effects such as wave action on different replicate days in the natural shore study, it is impossible to eliminate all possible confounds. To try to reduce such effects, a manipulative experiment was carried out on artificial substrata (marble rock slabs) on the shore at Cape d'Aguilar. The slabs created a simplified environment, with a flat rock surface and, prior to the setup of the experiment, no mucus trails. Slabs (300×300 mm) were placed flat on the shore at the mean high, high water level (mean of the maximum tidal height at spring tides). To prevent snails from escaping, Tree Tanglefoot gum (Tanglefoot Company, Michigan, USA) was placed around the perimeter of the slabs [16].

Ten replicates of three treatments were randomly assigned to 30slabs (∑*n* = 3×10 = 30). Ten slabs were shaded using a roof of aluminium foil wrapped around wire mesh (roof size 350×350 mm) supported 50 mm above the slab surface. The roof shaded the experimental area from direct sunlight and therefore lowered both temperature and desiccation. Ten slabs were procedural controls, consisting of a wire mesh roof (mesh size 7 mm) not covered in foil, therefore having the same roof frame but no shading. The final treatment was an unmanipulated open treatment (the control), with no roof and therefore subject to direct sunlight.

During low water, 7 *Echinolittorina malaccana* and 8 *E. radiata* (representative of the population density of each species on the shore, all ∼6 mm in shell length) were placed on the slabs and allowed to attach by their foot or mucus holdfast. During high water, waves (<100 mm in height) washed over the slabs for >1 h and the snails were observed to move. The tide began to retreat just prior to midday, so air temperatures were high (>35°C) when the plates dried and the snails stopped moving. Once all animals had stopped moving, the percentage of snails in aggregations on each slab was determined and slab surface temperature was measured with a K-type thermocouple (see [17] for details). Desiccation rates on each plate were estimated by measuring weight loss over 1 h of cotton wall balls soaked in water (1 cotton wool ball per plate) during mid-afternoon, after all snail movement had stopped.

### Computer simulation

#### Overview

The simulation was based on that used in [12] and consists of a number of virtual snails that move around a virtual shore, based on realistic and statistically accurate movement patterns. During the retreating tide, snails cease their foraging in the awash zone and move down the shore where, if chance encounters with previously laid mucus trails occur, they may choose to follow the trails. Also if encounters with other snails occur, then they may choose to aggregate with these snails, and consequently stop moving. Decisions are time-based: each decision to trail-follow or aggregate is more likely to occur the longer the simulation runs. This time base is a proxy for dryness of the rock (which becomes more dry the longer it is exposed to air after the tide retreats). A simulation can last for one or more tidal cycles, each of which consists of individuals moving up the shore, foraging in the awash zone, and moving back down the shore. At the end of the simulation, the percentage of snails in aggregations is calculated.

#### Implementation of the simulation

The simulated rocky shore (1 m^2^) was divided into a grid of 5×5 mm squares. Snails could move from one square to any of the eight neighbouring squares, giving an average distance between squares of ∼6 mm (accounting for the diagonal distance being further than the orthogonal), which was the length of an average snail. One timestep in the simulation was defined as the time taken for each snail to move from one grid square to a neighbouring grid square. The simulation normally lasted 400 timesteps for each simulated tidal cycle (equivalent to 3 h on a real shore). In most simulations, snails were prevented from moving off the top of the simulated shore by restricting upward movement directions, this typically only affected <5% of snails in any given simulation [12]. Snails moving off the side of the simulated shore rejoined on the opposite side, moving in the same direction. Snails were able to leave the bottom of the simulated shore, and if they did so in the final tidal cycle of the simulation, they were not included in further analysis. Snails leaving the bottom of the shore in other tidal cycles rejoined the simulation at the bottom of the grid at the start of the next tidal simulation, with their horizontal positions randomly determined.

Simulated snails were initially randomly located in the lower 100 rows of the grid. At the start of each tidal cycle, each individual moved up the shore with an initial bearing of 0°. At each timestep a new bearing was obtained by summing the previous bearing with a randomly-generated angle from a normal distribution (mean = 0°, SD = 10°). A bearing of 0° was also applied at timesteps 10, 20, 40 and 80 to ensure simulated patterns were similar to observed patterns in that the snails moved upshore [12]. Once in the top 40 rows of the grid (the position of the snails during slack water at high tide while they are foraging in the awash zone) the randomly-generated angle was changed to give a more tortuous movement pattern (mean = 0°, SD = 100°). As the tide retreated (from timestep = 250), snails following the tide down the shore were initially given a bearing of 180°, and then the bearing was again modified by a random angle (mean = 0°, SD = 10°). These bearings and angles mimicked real snail behaviour as they moved up and down the shore with the rise and fall of the tide [10], [12]. Patterns of simulated individual movement have previously been shown not to be significantly different from real snails' movement patterns in terms of length of the trail and fractal dimensions of the trail [10], [11].

**Figure 1:**
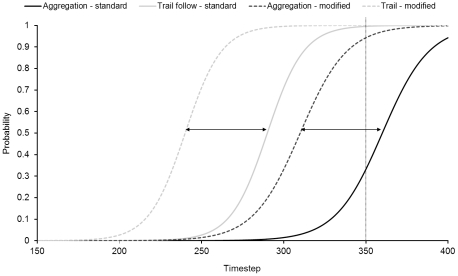
Probabilities of trail following or aggregating. The probability of trail following or aggregating given an encounter between two snails or a snail and a previously-laid mucus trail. Right of the arrows indicates ‘standard’ parameters for equation 1 as in [12]: trail following *a* = 0.9 , *b* = 290, aggregation *a* = 0.7, *b* = 360. Left of the arrows indicates modified parameters used to indicate higher levels of desiccation stress: trail following *a* = 0.9, *b* = 240, aggregation *a* = 0.7, *b* = 310. The vertical line indicates when snails stop moving in hotter, more desiccating conditions.

Decisions were made when snails encountered other snails, and when snails encountered the mucus trails of other snails. To simulate a decision, a random number (from a uniform distribution between 0 and 1) was compared to the probability threshold, *y*, which increased sigmoidally with the time of the simulation (to create a proxy for dryness of the rock):
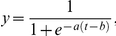
where *t* is the timestep, normally between 1 and 400, and values of parameters *a* and *b* control the probability of the decision occurring at a given timestep. These parameters were modified depending on the simulation (see Figure 1). If the random number was lower than the probability, then the decision to aggregate or trail-follow took place. The likelihood of trail following or aggregating, therefore, increased with the advancing number of timesteps of the simulation, but still depended on chance encounters with other snails or trails. Although aggregated snails were not moving, they could take part in further aggregation decisions if other snails encountered them – this may result in these aggregations increasing in size.

To simulate the observed effects of increased desiccation, the number of timesteps in some of the simulations was reduced from 400 to 350. This simulated the behavioural response of snails which stop moving earlier in relation to increased desiccation stress and the drying of the rock surface. This reduction in time is indicative of the shorter foraging time experienced under hotter conditions, as measured in different treatments in the artificial environment experiment (see Results). To simulate differences in individual level behaviour as a result of increases in desiccation stress, in addition to the changes in the total number of timesteps, the behavioural rules of aggregation formation and trail following were also altered (Figure 1). This allowed decision outcomes of ‘aggregate’ and ‘follow mucus trails’ to occur with greater probability earlier in the simulation, since we predict that decisions made by the snails (although modelled as time-based) are related to the dryness of the rock surface [11]. Previous observations of snail movement at different rock surface temperatures indicated no change in speed of snail movement with temperature until just prior to the point when the snails stopped moving [10].

To provide simulation results to compare to the shore study, 1 m^2^ of shore was simulated with the same density of snails as recorded from the real shore (112 individuals m^−2^, based on mean snail densities in quadrats analysed in this study). Persistence of mucus trails has previously been shown to be important in determining the spatial and temporal patterns of snails on the shore and degradation of mucus trails during tidal cycles was modelled as per [12]:
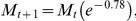
where *M* is the amount of mucus present in each 5×5 mm grid square. To ensure the effects relating to persistence of mucus trails were incorporated into the simulation, each run of the simulation was repeated over three tidal cycles before results were collected, thus allowing simulation of mucus trails laid on the shore during previous movements of snails. A randomly selected area (a 500×500 mm quadrat – equal in area to the shore study) of the simulated shore was then investigated. Since we randomly selected quadrats on the shore, crevice distribution varied between replicate quadrats, and, as crevice distribution can affect the proportion of snails aggregating (although not significantly, [12]), we simulated all shores to have no crevices. Thus, our simulation results would not be expected to show exact matches to the observed data from real snails, but should be indicative of trends caused by changing physical factors.

To compare the simulation with the semi-artificial environment study, we simulated a shore of 300×300 mm – the same size as the marble slabs used in the semi-artificial environment study. We prevented simulated snails moving outside these dimensions by preventing movement off the edge until a new direction within the 300×300 mm area was established, as would occur with real snails that were restricted to moving solely within the artificial environment. Since the snails were already in the awash zone when first immersed, and could not move fully down the shore with the retreating tide, the times of the simulations were restricted to 200 timesteps (from timestep 150 to timestep 350) for the low desiccation (shaded) treatment and 175 timesteps (150 to 325) for the open treatment.

For both simulations of the natural shore study and the semi-artificial environment study three sets of simulations were conducted:

1. simulations over the full duration of the model (400 timesteps for the shore study and 200 for the semi-artificial environment study);
2. simulations over the reduced length model (350 timesteps for the shore study and 175 for the semi-artificial environment study) but with no changes to the behavioural rules of the individuals;
3. simulations over the reduced length model (350 timesteps for the shore study and 175 for the semi-artificial environment study) but with changes to the individual's behavioural rules as indicated in Figure 1.

## Results

### Natural shore study

**Figure 2:**
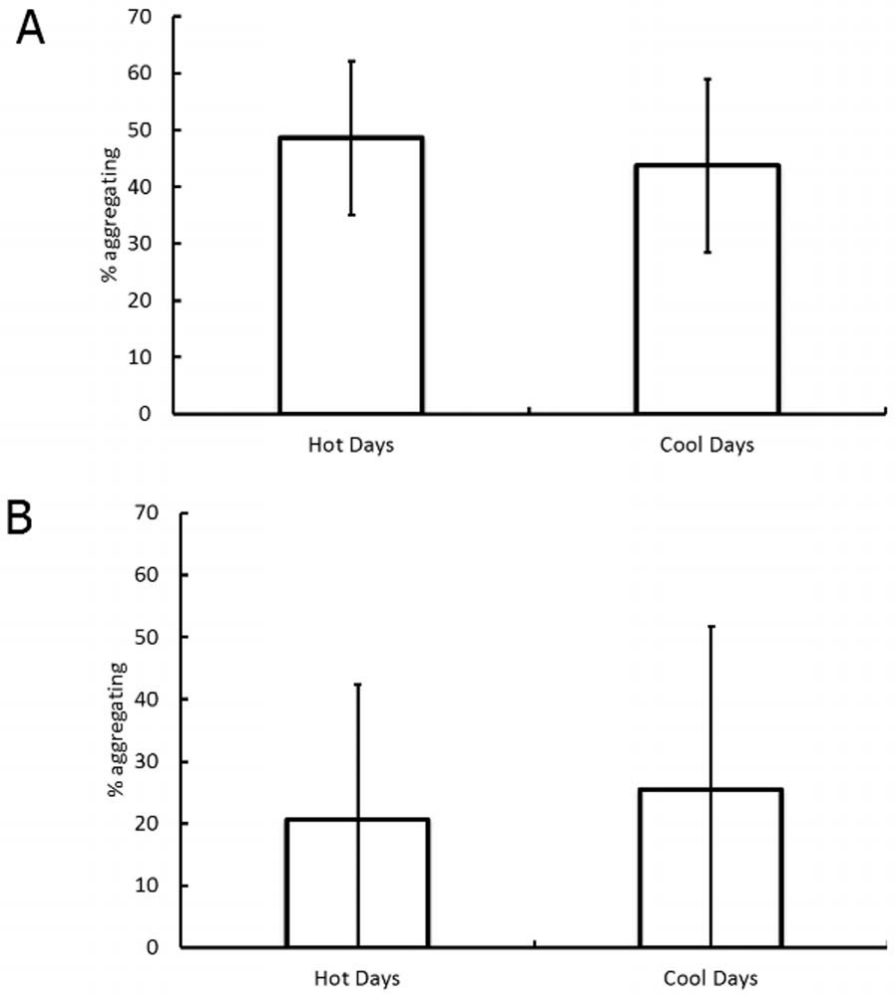
Aggregations on Hot and Cool days. Mean (± SD, n = 30) percentage of snails aggregating on Hot Days (>35°C) and Cool Days (<28°C) at (a) Cape d'Aguilar and (b) Wah Fu.

Desiccation rate differed significantly between the different temperature conditions (mean ± SE n = 15, desiccation rate = 71±1.4% h^−1^ during Hot Days compared to 51±0.7% h^−1^ during Cool Days), although significant differences were also observed between replicate days within the same conditions (Table 1). There were, however, no significant differences in the percentage occurrence of aggregation behaviour between Hot or Cool Days or replicate days within conditions at Cape d'Aguilar (Table 1; Figure 2a) or Wah Fu (Table 1; Figure 2b).

**Table 1:** Summary of comparisons using nested ANOVA to determine variation in desiccation of cotton wool balls and aggregation behaviour of littorinid snails during different environmental conditions (Factor = Temperature, fixed with 2 levels, Hot or Cool Days) on three replicate occasions (Factor = Day, random with 3 levels).

Comparison	Shore	Factor	*F*	d.f.	*p*
Desiccation of cotton wool balls at different temperatures	Cape d'Aguilar	Day(Temperature)	2.50	8, 20	**0.043**
		Temperature	96.00	1, 20	**<0.001**
Aggregation behaviour at different temperatures and low wave action	Cape d'Aguilar	Day(Temperature)	0.40	4, 54	0.806
		Temperature	3.99	1, 54	0.166
Aggregation behaviour at different temperatures and high wave action	Wah Fu	Day(Temperature)	0.39	4, 54	0.390
		Temperature	5.22	1, 54	0.084

Values in bold indicate significant differences between treatments (*p*<0.05). For all comparisons: Levene's test for homogeneity of variance *p*>0.05, not significant.

### Semi-artificial environment study

**Figure 3:**
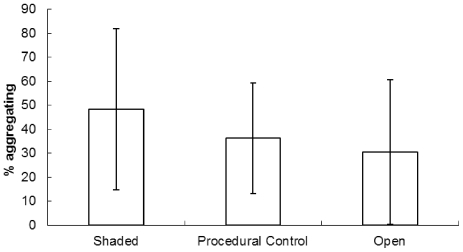
Aggregations in semi-artificial environments. Mean (± SD, n = 10) percentage of snails aggregating in semi-artificial environments (marble rock slabs) under three different treatments (Shaded, procedural control and open).

On artificial slabs, both temperature and desiccation rate were significantly lower (by ∼20°C and 30%) in the shaded as compared to the open or procedural control treatments (Table 2). No significant difference was, however, found between the percentages of snails aggregating in different treatments (Table 2), although the mean percentage of snails aggregating was higher, by almost 20%, in the shaded treatment as compared to the open treatment, and >10% higher in the shaded treatment than in the procedural control (Figure 3). Snails stopped moving in the open treatments ∼15–20 min before the covered treatments, which equates to changes in foraging time of ∼50 model timesteps.

**Table 2:** Summary of comparisons using ANOVA and subsequent SNK tests to determine differences between surface temperature, desiccation and sheltering behaviour of littorind snails on marble rock slabs with different treatments (open to full sunlight, shaded with a roof and a procedural control).

Comparison	*F*	d.f.	*P*		SNK tests	
Temperature	210.8	2, 27	**<0.001**	Open =	Procedural control >	Shaded
Desiccation	79.4	2, 27	**<0.001**	Open =	Procedural control >	Shaded
Aggregation behaviour	0.98	2, 27	0.388			

Values in bold indicate significant differences between treatments (*p*<0.05). For all comparisons: Levene's test for homogeneity of variance *p*>0.05, not significant.

### Computer simulation

**Figure 4:**
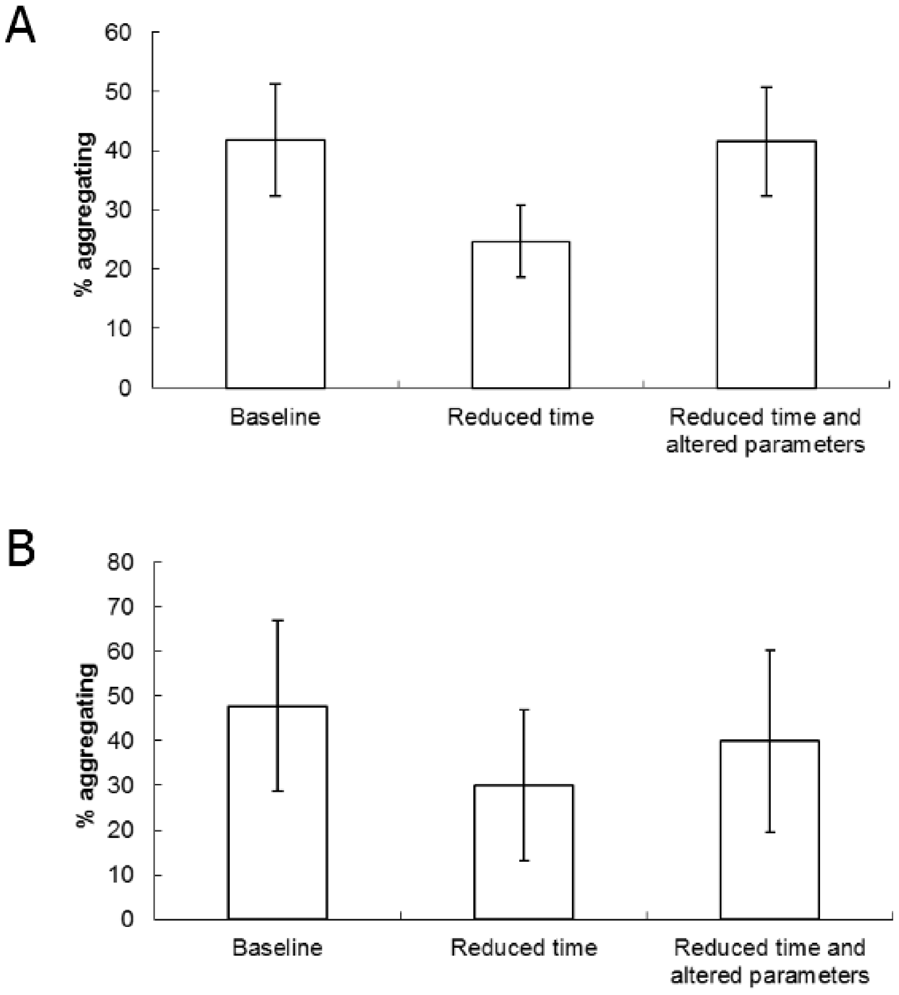
Aggregations in simulated experiments. Mean (± SD, n = 500 or 2000) percentage of snails aggregating in (a) simulated shore experiments and (b) simulated semi-artificial environment experiments.

Simulations of real shores (equivalent to the natural shore study observations detailed above) were run 500 times for each treatment to ensure a reliable estimate of the true mean value (SE<0.5% of mean). The initial simulation predicted a mean of 41.8% of snails in aggregations (Figure 4a). This percentage decreased to 25.1% when the time of the simulation was reduced to 350 timesteps, but increased to levels similar to those initially recorded (42.1%) when both the time and decision parameter values were altered (Figure 4a).

The simulations of the slab experiments were run 2000 times for each treatment to ensure a reliable estimate of the true mean value (SE<0.5% of mean). The baseline conditions, in this case indicative of the coolest conditions (shaded treatment), showed a mean of 48% of snails in aggregations (Figure 4b). Decreasing the time of movement resulted in a large reduction in the proportion of aggregating snails (to 30%). Altering the parameters of the behavioural rules (*a* and *b*), as well as reducing the time of movement, gave an intermediate mean value of 40% of snails in aggregations (Figure 4b).

## Discussion

During hot, desiccating conditions, individual-level snail behaviour changes from that exhibited in cooler, less desiccating conditions. The time snails spend moving decreases, probably because the rock surface dries faster [11]. This should give snails less time to locate other individuals and form aggregations, therefore fewer or smaller aggregations should be formed, i.e. an individual-level change in behaviour has a direct effect on self-organised behaviour [11]. This appears to be a sub-optimal behaviour, as aggregation reduces desiccation [10], and the consequences of not aggregating on highly desiccating days should be greater than on cooler days. This theory is supported by results from the semi-artificial environment studies and computer simulations of those studies, indicating there are clear differences in the self-organised behaviour as a result of behavioural changes at the individual level in this situation. However, results from fully natural environments showed no change in the collective behaviour, despite differences in desiccation between shaded and unshaded, and hot and cold days, being broadly similar. This was true of directly recorded results from the shore, as well as simulations of the snails from the natural shore environment (essentially the inclusion of mucus trails that were persistent between tidal cycles). The lack of change in the percentage of snails aggregating demonstrates that in natural environments, self-organised behaviours can be robust to changes in individual-level behaviour.

Clear differences were found between the two natural shore sites investigated, with Cape d'Aguilar having a much higher proportion of aggregating snails than Wah Fu. However, this is likely to be due to crevice abundance at the two sites (Wah Fu having numerous small crevices [10]), rather than due to differences in behaviour of the snails or other environmental variables. Presence of ample small refuges have been shown to reduce aggregation behaviour [18]. Hence, in this study, the lack of relative change between environmental conditions is of more interest than the absolute values of aggregation at different sites.

In evolutionary terms, self-organisation, at least in the case of littorinid snails, can overcome the need for complex sensory and cognitive systems that deal with changing environments, which can be costly to develop [19]. While constraints in sensory and cognitive systems have previously been shown to produce non-perfect behaviours (those that do not correspond to a predicted optimal value, such as sex ratio [20]–[22]), it may be that in some cases, simple, low complexity sensory systems are able to perform better in self-organising systems than in systems where no self-organisation occurs. In this study, by simply sensing rock dryness, and having behaviours governed by this single parameter, foraging time will be increased where possible (i.e. in cooler conditions) and aggregation levels can remain high in both cool and hot conditions. Essentially, self-organisation allows the snails to avoid some of the developmental costs of complex sensory systems and still optimise both their feeding and sheltering behaviours.

In terms of application to other studies of self-organising systems, the results suggest that tests of the effectiveness of collective behaviour could be limited if they are conducted in simplified environments (such as the laboratory), or in computer simulations with little regard to the natural environment. This supports much work, from sub-organismal, neuroethological studies to community or global level studies [e.g. 23]–[25], suggesting that an integrated understanding of interactions of organisms in their environment is important in elucidating the true functional or adaptive benefits of behaviours and other ecological processes.
